# The Virchow Banquet

**Published:** 1893-03-25

**Authors:** 


### The Virchow Banquet.

The great questioners of the present age are the members of the medical profession. Other departments of science may show here and there an inquirer; but every modern medical man, who is at the same time intelligent and honest, is above all things an ardent explorer in the regions of newer and larger truth. Professor Rudolf Virchow, the pioneer of the modern pathology, was honoured on Thursday evening with those honours which the British Teuton most deeply and intimately appreciates, the significant honours of the dinner-table. When your Briton, of his own motion, shakes hands with a man, the act is indicative of much; but when he goes a long way out of his beaten track for the purpose of voluntarily dining with him, he does the utmost that he can do. Years ago every scientific honour which British medicine has to bestow had been bestowed upon Professor Virchow. It was not until last week, at the ripe age of seventy-two, that he attained to the grand climax of a dinner.

What is the distinguishing mark of the great Berlin Professor as it reveals itself to those who look for it? He is a "plain, blunt man," of careful industry and large intelligence, who honestly speaks what he clearly sees. He will not go beyond the record. That is science. Other eminent men of science have gone beyond the record. Darwin was notorious for taking the bit in his teeth and prancing off. Darwin was the prince of speculators. Tens of thousands followed in his train who were not able to see that he had taken the bit between his teeth. Virchow is to-day a better trusted man than Darwin ever was.

In listening to Virchow, Huxley, and Paget last week, the younger men of the present generation must have felt as if the ghosts of certain great ones had suddenly stepped from out the pages of our scientific text-books. We who have from our professional infancy been familiar with "irritability," "contractility," and the "cell pathology" had not realised that in the very next generation preceding our own "irritability" and "contractility," in their physiological significance were new-comers in the region of scientific ideas; new-comers which aroused suspicion and antagonism, and which had to fight their way inch by inch to the secure position of accepted truths, exactly as an ambitious man of real capacity has to secure his ground step by step until everybody acknowledges his right to recognition and honour. There was something of an actual apocalypse in all this If it taught us nothing else it taught us how very young a giant scientific medicine really is; and how easy it may be for such a giant, until he has worked out a more experienced temper, to make foolish and desperate lunges in all directions, which may be productive of almost as much harm as good.

What is the chief characteristic of the "cell pathology" of which Virchow is the admirable father and expounder? In plain words, it may be called "the pathology of fact as opposed to the pathology of fancy." There were certain Eastern monks in the long ago who sought to evolve deeper religious knowledge and truth by the persistent contemplation and study of their own navels. Our pathological ancestors were, if possible, almost more inconsequent than even they. The study of one's own navel is really a form of external observation, even if not a very profitable one. But the pathologists of our great grandfathers' times sought to bring pathology from their inner consciousness or from the clouds; anywhere and everywhere rather than from the continuous and intelligent observation of living things in their methods of work and life. When you want to know about a thing you must look at the thing itself. This would seem to be a rule so obvious that not even a fool could miss it. Nothing of the kind, said our forefathers: when you want to ascertain all about a man's liver you must contemplate the stars; if it be his heart you wish to understand you had better consult the inner recesses of your personal consciousness.

It was a merry meeting at the Metropole, and did great credit to its organiser, Mr. Jonathan Hutchinson, F.R.S. Lord Kelvin showed himself almost as good a pathologist as an engineer and an electrician. He made an admirable chairman. Professor Huxley was very unusually solemn. He was, however, looking well; and an occasional dilatation of the nostrils, as he sounded his rather laboured periods, made one aware that he was still a "war-horse" who "smelt the battle from afar." Sir James Paget was, as he always is, Sir James Paget: honest, gentle, conciliatory, a charming gentleman. But even he was almost roused to ire as he thought of the ages and generations wasted which might have been turned to infinite account if "observation" had prevailed in our fathers' days instead of the "reflection" which never "reflects." Sir Andrew Clark rose to complain of "broken vows" on Mr. Hutchinson's part. He had stipulated that he should not be called upon to speak; and Mr. Hutchinson had apparently accepted the stipulation. Yet here was a toast put into his mouth! How men of such scientific eminence as Mr. Hutchinson can reconcile such infractions of solemn contracts with the doctrine of "facts, evermore facts, and nothing but facts," it passes the wit of man to conceive. Probably the great surgeon had forsaken for a moment the severely literal goddess "Observation" and indulged in a reprehensible flirtation with her more vivacious sister "Reflection." He had made an excursus into his "inner consciousness," which for a doctor was painfully wrong. But notwithstanding previous "stipulations" and "unpreparedness," Sir Andrew Clark made an eloquent speech, and brought down the house in a way one would have thought impossible if one had formed one's opinions about doctors solely from the experience of the consulting-room.

Professor Virchow, the most distinguished of specialists, has sounded the knell of hyper-specialism. He is a pathologist, an anthropologist, a student of Homer, and a politician: it is said also that he has an intimate knowledge of the varieties and values of farmyard manures. In all these various walks of life he leads and does not follow, or follows as only a leader can. There are in reality only two legitimate specialisms in medicine. A man is a physician, or he is a surgeon. Indeed, the true physician is a surgeon in all but his familiarity with operative procedures; and the competent surgeon is a physician practically without any exception at all. The subdivision of the medical art, and its breaking up into a thousand-and-one microscopic and miserable fragments is, as Sir Dyce Duckworth has said, a "plague, a degradation," and an absurdity. Professor Virchow is the last man in the world to claim any other specialty and distinction than that of honest, clear-sighted industry. If such a man as he can serve his country as a professor of pathology and a politician, and if he can find time also to lend the world a guiding hand in anthropology and agriculture, to say nothing of the Iliad and the Odyssey, surely our one-eyed specialists, who devote their strenuous feebleness exclusively to "flatulalgia," "digititis," or the like, will pluck up manliness enough to be ashamed of themselves. This grand old man of pathology has set an example which we must all take to heart.

Professor Virchow is, as we have said, the father of the cellular pathology. What, in a word, is the nature of the revolution which that pathology has made in our conceptions of living forms? This question may be most fittingly answered in Virchow's own eloquent words: "Since the cellular constitution of plants and animals has been proved, and since cells have become recognised as the essentially living elements, the new science of biology has sprung up. It has not brought us the solution of the ultimate riddle of life, but it has provided concrete material, anatomical objects for investigation, the properties, the actions, and the passions of which we can analyse. It has put an end to the wild confusion of fantastic and arbitrary notions such as I have just mentioned; it has placed in a strong light the immeasurable importance of anatomy, even in the most delicate conditions of the body; and, lastly, it has made us aware of the close similarity of life in the highest and lowest organisms, and has thus afforded us invaluable means for comparative investigation." In a word, in matters biological and medical, the cellular pathology "has made all things new."

